# Application of a machine learning method to whole brain white matter injury after radiotherapy for nasopharyngeal carcinoma

**DOI:** 10.1186/s40644-019-0203-y

**Published:** 2019-03-25

**Authors:** Xi Leng, Peng Fang, Huan Lin, Chunhong Qin, Xin Tan, Yi Liang, Chi Zhang, Hongzhuo Wang, Jie An, Donglin Wu, Qihui Liu, Shijun Qiu

**Affiliations:** 1 Medical Imaging Center, The First Affiliated Hospital of Guangzhou University of Traditional Chinese Medicine, Guangzhou, Guangdong 510405 People’s Republic of China; 2 Department of Psychology, The Fourth Military Medical University, Xi’an, Shaanxi 710032 People’s Republic of China; 3 Department of Radiology, Zhujiang Hospital of Southern Medical University, No. 253, Gong Ye Da Dao Zhong, Guangzhou, Guangdong 510280 People’s Republic of China

**Keywords:** Diffusion tensor imaging, Radiation injuries, Nasopharyngeal carcinoma

## Abstract

**Background:**

The purpose/aim of this study was to 1) use magnetic resonance diffusion tensor imaging (DTI), fibre bundle/tract-based spatial statistics (TBSS) and machine learning methods to study changes in the white matter (WM) structure and whole brain WM network in different periods of the nasopharyngeal carcinoma (NPC) patients after radiotherapy (RT), 2) identify the most discriminating WM regions and WM connections as biomarkers of radiation brain injury (RBI), and 3) supplement the understanding of the pathogenesis of RBI, which is useful for early diagnosis in the clinic.

**Methods:**

A DTI scan was performed in 77 patients and 67 normal controls. A fractional anisotropy map was generated by DTIFit. TBSS was used to find the region where the FA differed between the case and control groups. Each resulting FA value image is registered with each other to create an average FA value skeleton. Each resultant FA skeleton image was connected to feature vectors, and features with significant differences were extracted and classified using a support vector machine (SVM). Next, brain segmentation was performed on each subject’s DTI image using automated anatomical labeling (AAL), and deterministic white matter fiber bundle tracking was performed to generate symmetrical brain matrix, select the upper triangular component as a classification feature. Two-sample t-test was used to extract the features with significant differences, then classified by SVM. Finally, we adopted a permutation test and ROC curves to evaluate the reliability of the classifier.

**Results:**

For FA, the accuracy of classification between the 0–6, 6–12 and > 12 months post-RT groups and the control group was 84.5, 83.9 and 74.5%, respectively. In the case groups, the FA with discriminative ability was reduced, mainly in the bilateral cerebellum and bilateral temporal lobe, with prolonged time, the damage was aggravated. For WM connections, the SVM classifier classification recognition rates of the 0–6, 6–12 and > 12 months post-RT groups reached 82.5, 78.4 and 76.3%, respectively. The WM connections with discriminative ability were reduced.

**Conclusions:**

RBI is a disease involving whole brain WM network anomalies. These brain discriminating WM regions and WM connection modes can supplement the understanding of RBI and be used as biomarkers for the early clinical diagnosis of RBI.

## Background

Nasopharyngeal carcinoma (NPC) is a common cancer with a high incidence among head and neck malignant tumours in southern China [1]. Radical radiotherapy (RT) is the first-choice treatment. However, RT may cause radiation brain injury (RBI), which is the most serious complication [2]. RBI can be roughly divided into three stages according to the time of occurrence: the acute reaction period, the early delayed radiation period and the late delayed radiation period. Injuries of the late delayed radiation period are often irreversible [3]. RT-induced cognitive impairment significantly decreases the quality of life of NPC patients [4–6]. RBI is most prominent in cognition, memory, and emotion [7]. How to detect RBI early, before conventional CT and MRI images appear abnormal, is the key problem to improve the quality of life and prognosis of NPC patients.

Diffusion tensor imaging (DTI) is the only technology that can evaluate the microstructural and morphological changes of white matter (WM) fibres in vivo and has been widely used in the study of the histomorphology and pathology of the central nervous system (CNS) associated with RT [8, 9]. In general, the damage to WM in RBI is greater than that to grey matter (GM). The blood supply of the WM is relatively scarce. After exposure, the blood vessels are damaged, and ischaemic necrosis easily occurs [10]. Therefore, most of the studies related to RBI are focused on WM, for which decreased fractional anisotropy (FA) values indicate loss of WM integrity. FA was one of the most commonly DTI parameter, which was used to quantify the degree of contrained water diffusion along the axons and myelin. However, the exact physiopathology of RBI is still unknown.

Previous studies are traditionally based on group-level comparisons [11, 12]. Due to the different research techniques and sample sizes, different studies often yield inconsistent or even contrary results. In contrast, the machine learning method has been used widely in brain image analysis, can extract additional information and stable patterns from brain image data, and can identify or distinguish patients from normal persons at the individual level, identifying biomarkers based on neuroimaging data [13–15]. We hypothesized that based on the whole brain DTI data, machine learning method could separate NPC patients post-RT from normal persons at the individual level and further explore the pathophysiological mechanism of RBI. FA value and WM integrity might be able to discriminate patients of different month, and these FA value and WM integrity would be selected as the features in classification. To the best of our knowledge, the present study is the first to use DTI, tract-based spatial statistics (TBSS) and machine learning methods to examine dynamic changes in the whole brain WM microstructure and WM network situation, selecting the most discriminating WM regions and WM connections in NPC patients after RT.

## Methods

### Patients

The present study included 77 patients (54 males, 23 females; aged between 25 and 59 years; mean age, 45 years) with pathologically confirmed NPC, and the normal control group contained 67 cases. All patients underwent fractionated RT for the first time with three-dimensional conformal and intensity-modulated techniques (total dose/fraction dose/exposures, 66–74 Gy/1.8–2.0 Gy/30–35 times). Prior to MRI examinations, it was validated that the patients exhibited no intracranial tumours or intracranial invasion. Patients with high blood pressure, diabetes, heart disease, WM degeneration or vascular lesions were excluded.

Normal subjects constituted the control group, the same exclusion criteria that applied to RT subjects were applied to controls (i.e. hypertension, diabetes, heart disease, WM degeneration, vascular lesions). Traditionally, according to time of completion of RT, neurological impairment induced by RT can be described in terms of acute injury (few days to weeks), early delayed injury (1 to 6 months) and late delayed injury (> 6 months) [16]. So in our study, post-RT patients were divided into three groups according to the stage of RBI: acute reaction period, early delayed radiation period and late delayed radiation period: Group 1 (0–6 months post-RT, *n* = 30); group 2 (> 6–12 months post-RT, *n* = 20); and group 3 (> 12 months post-RT, *n* = 27). No statistically significant differences were identified among the groups according to age or sex. Demographic and clinical data are presented in Table 1. The present study was approved by the Institutional Review Board and was conducted under strict adherence to the Privacy Rules of The Health Insurance Portability and Accountability Act. All individuals included were fully informed of the purpose, methods and precautions of the trial, and written informed consent was obtained from all participants.

**Table 1:** Demographic and clinical data

Group	N	Sex		Age (year)	Education (year)
		Female	Male		
Control	67	19	48	44.28 ± 7.53	10.46 ± 2.74
I (0–6 m)	30	10	20	43.00 ± 9.27	10.40 ± 3.31
II (6–12 m)	20	5	15	46.40 ± 9.72	10.55 ± 3.30
III (> 12 m)	27	8	19	46.78 ± 8.96	9.93 ± 3.32
*F* or *χ*^2^ Value		0.290		1.91	1.70
*P*-value		0.590		0.170	0.196

Group I, II, III: NPC Patients examined 0~6, 6~12, and > 12 months after radiotherapy, respectively. The F or *χ*^2^ value and *P*-value are from ANOVA or Pearson’s *χ*^2^ test

### Image acquisition

MRI data were acquired using a 3.0 T clinical scanner with an eight-channel head coil (SIGNA EXCITE; GE Healthcare, Chicago, IL, USA). The routine MRI brain protocol included axial T1-weighted images [repetition time (TR), 600 ms; echo time (TE), 15 ms], T2-weighted images (TR, 5200 ms; TE, 140 ms) and T2-weighted fluid attenuated inversion recovery (TR, 9000 ms; TE, 120 ms; inversion recovery, 2100 ms). DTI scans were performed, employing a single-shot echo-planar imaging sequence and an array spatial sensitivity encoding technique with the following parameters: TR, 12,000 ms; TE, 75.5 ms; field of view (FOV), 24 × 24 cm; matrix, 128 × 128; slice thickness, 3 mm (no inter-slice gap); number of excitation, 1; and flip angle, 90°. Images were collected along 25 non-collinear diffusion gradient directions, with a b-value of 1000 s/mm2 and one set of null images with a b-value of 0 s/mm^2^.

### Image analysis

Images obtained in DICOM format were first converted to ANALYZE. The four-dimensional diffusion tensor image was then aligned with the first volume using McFlirt (FSL tool) (FMRIB analysis set; Oxford University, Oxford, UK) to eliminate head motion errors. Then, the eddy current-induced distortion of the aligned diffusion tensor image was corrected using affine registration with eddy current correction (FSL tool) (Oxford University). After completing these preprocesses, the obtained images were brain extracted using the FSL Brain Extraction Tool (BET) (Oxford University) and diffusion tensor models were fitted at each voxel using DTIFit (FMRIB Software Library’s Diffusion Toolbox) (Oxford University) to generate images of FA. Using TBSS, voxel-wise cross-participant comparisons were made between the FA profile of the NPC patient and the control participants to identify discrete regions of WM abnormalities. First, the target image was determined by aligning each participant’s FA image with each of the other images to determine the most representative theme. The target image was then normalized to the MNI 152 standard space using affine transformation. All other participants were then first aligned to the target image and then aligned to a 1 × 1 × 1 mm MNI 152 space, and nonlinear registration was achieved using FNIRT (FSL tool). This process created an average FA skeleton that represents the centre of all regions that were common to the group. Aligned FA data (NPC patients and control participants) from all individual participants were projected onto the FA skeleton, and the resultant data were used for voxel classification.

#### Brain segmentation

Brain segmentation is an important step in network construction. In this paper, the brain image is segmented by using the automated anatomical labeling (AAL) method to cover each subject’s DTI brain. This method divides the brain into 116 small brain regions, including 90 brain regions and 26 brain regions of the cerebellum. First, the T1 image is registered to the b0 image by linear registration of rotation and translation in the diffusion tensor space. The registered T1 image is then registered to the T1 image in the standard MNI space, and the resulting transformation matrix is inverted, and then the AAL template is transformed from the MNI space to the diffusion tensor space using the inverse matrix. This gives the AAL template for each participant.

#### White matter fiber bundle tracking

Deterministic white matter fiber bundle tracking using the FACT algorithm in TrackVis software (http://www.Trackvis.org).

#### Network construction

Combine the a and b results to generate a brain connection matrix. Each brain region is treated as a region of interest (ROI), called a node, so node v can be described as ROI(v). The connection between the two nodes ROI(v) and ROI(u) is defined as the edge e = (v, u). We define the weight w(e) of each edge e as the number of fibers between ROI(v) and ROI(u). So for each participant, we get a symmetric 116 × 116 matrix. To remove the diagonal component, we choose the upper triangular component (6670 elements) as the classification feature.

### Classification

For FA value, the FA skeleton image was first connected to the feature vectors and combined into rows in the large feature matrix. We extracted the FA skeleton matrix from the large feature matrix, leaving a non-zero feature. However, the remaining non-zero feature dimensions are still too high for direct classification, and due to registration errors and image noise, the distinguishing features are masked by useless features. Reducing the dimension of the feature space not only speeds up the calculation, but also improves the classification performance. And this study used a simple and effective two-sample t-test to select the most discriminating features.

In machine learning methods, feature selection is usually accompanied by feature reduction. As an unsupervised nonlinear dimensionality reduction algorithm, local linear embedding (LLE) can obtain low-dimensional embedding while maintaining the intrinsic structure of data due to its nonlinearity, geometric intuition and computational feasibility [17]. In this study, LLE was used to reduce the dimension of the feature space to a more manageable level. In the classification section, we chose support vector machines (SVMs) as our classification algorithm because they are flexible for overfitting, allow for the extraction of feature weights, and are often used for neuroimaging studies and often used for neuroimaging studies [18].

For brain WM connections, we first used a two-sample t-test to extract features with significant differences between groups. Then, using local linear embedding (LLE) for nonlinear feature extraction, the feature dimension is reduced to a more controllable degree. Finally, the support vector machine (SVM) is used for classification.

We used the leave-one-out cross validation (LOOCV) strategy to estimate the generalization rate (GR) of the SVM classifier in this study due to the limited sample size, assuming a total of N patients. In each fold of the LOOCV, N-1 patients are selected to train the SVM classifier, and the remaining patient is left to test the classifier. In each fold of LOOCV, we used a two-sample t-test to select the most significantly different D features for N-1 trained patients first. Then, LLE was performed to reduce the feature space dimension from D to d. The results were used to train the SVM classifier, and the remaining patient was used to evaluate classifier performance by comparing the classification results to the ground truth class label. Since there are N samples, the classifier is trained and tested N times in the LOOCV strategy. Based on the results of LOOCV, sensitivity (SS), specificity (SC) and GR are used to quantify the performance of the classifier. SS represents the proportion of patients correctly classified, and SC represents the proportion of controls that are correctly classified. The overall proportion of correctly classified samples is represented by GR. Using the SVM prediction score for each participant as a threshold, a receiver operating feature (ROC) curve was constructed to further estimate the performance of our classifier. In addition, GR was used as a statistic to apply a permutation test to assess the statistically significant level of the observed classification accuracy. The class labels of the training data are randomly permuted, and then cross-validate each group of label-permuted data. The entire permutation process was repeated 10,000 times.

## Results

**Fig. 1:**
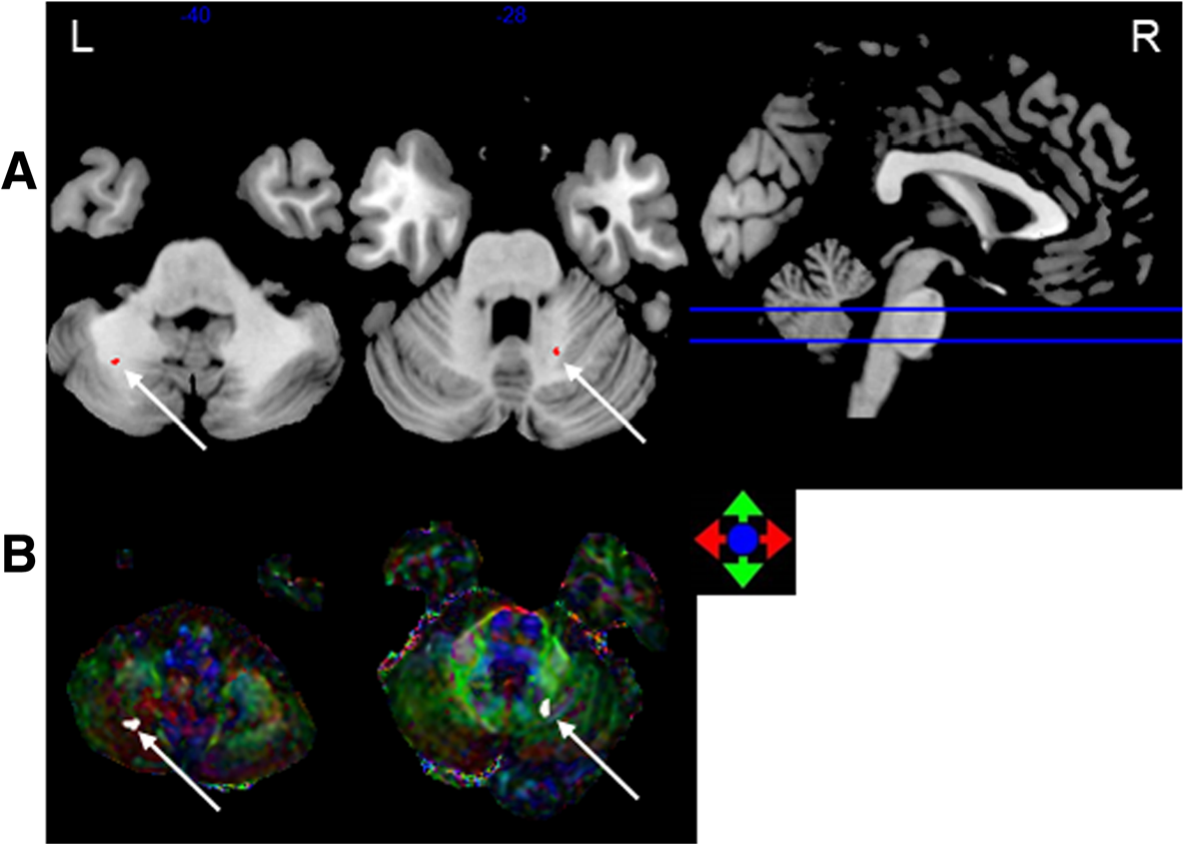
The most discriminating voxels for the classification of 0–6 months post-RT versus control; the image is the cutaway view: **a** displayed on the mean group fractional anisotropy (FA) map, the abnormal regions are shown in red; **b** FA map displayed in color (Red represents the left and right direction, green represents the up and down direction, and blue represents the front and rear direction). The left side of the brain is on the left side of the image. L = left, R = right

**Fig. 2:**
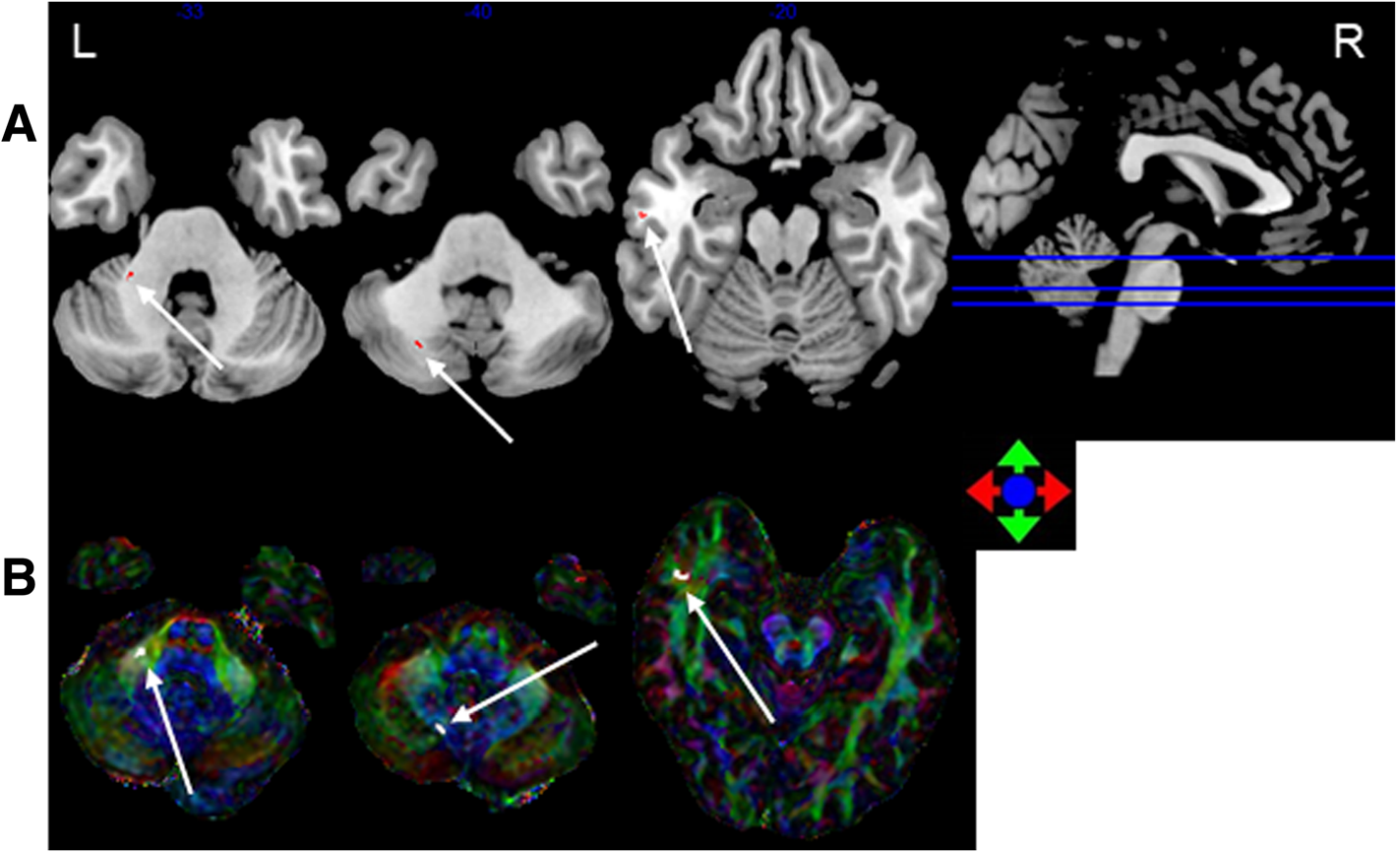
The most discriminating voxels for the classification of 6–12 months post-RT versus control; the image is the cutaway view: **a** displayed on the mean group fractional anisotropy (FA) map, the abnormal regions are shown in red; **b** FA map displayed in color (Red represents the left and right direction, green represents the up and down direction, and blue represents the front and rear direction). The left side of the brain is on the left side of the image. L = left, R = right

**Fig. 3:**
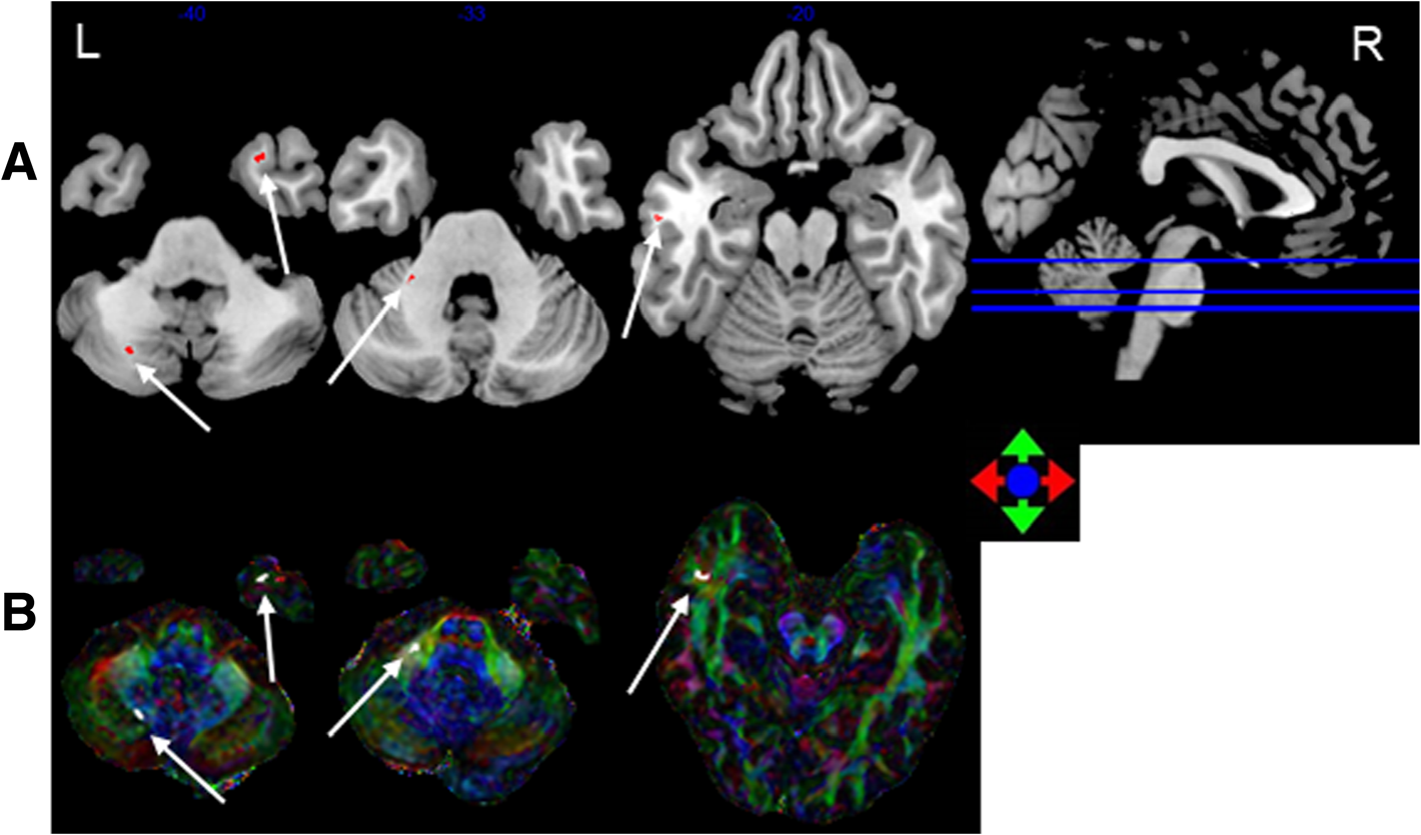
The most discriminating voxels for the classification of > 12 months post-RT versus control; the image is the cutaway view: **a** displayed on the mean group fractional anisotropy (FA) map, the abnormal regions are shown in red; **b** FA map displayed in color (Red represents the left and right direction, green represents the up and down direction, and blue represents the front and rear direction). The left side of the brain is on the left side of the image. L = left, R = right

FA. The accuracy of classification between the 0–6, 6–12 and > 12 months post-RT groups and the control group was 84.5, 83.9 and 74.5%, respectively (Table 2). Compared with the control group, in the 0–6 months post-RT group, the FA values in the brain regions with discriminative ability were reduced, mainly in the bilateral cerebellum, including Cerebelum_7b_L and Cerebelum_Crusl_R (Johns Hopkins White Matter Tractography atlases, JHU:Cerebellum white matter). The FA values of the 6–12 months post-RT group in the brain regions with discriminative ability were reduced, and the main area was located in the left temporal lobe WM and the left cerebellum, including Cerebelum_Crusl_L, Cerebelum_8_L (JHU:Cerebellum white matter), and Temporal_Mid_L (JHU:Middle temporal white matter). The FA values of the > 12 months post-RT group in the brain regions with discriminative ability were reduced, mainly in the bilateral temporal lobe and cerebellum, including Cerebelum_Crusl_L, Cerebelum_8_L(JHU:Cerebellum white matter), Temporal_Mid_L and Temporal _Pole_Sup_R (JHU:Middle temporal white matter) (Figs. 1, 2, 3 4 and 5). With prolonged time after RT, the damage was aggravated, and the number of brain areas that had the most discriminative ability increased gradually.

**Table 2:** SVM classification results of FA

	GR	SS	SC	Permutation test
0–6 m vs. controls	84.5%	86.7%	83.6%	*P* < 0.0001
6–12 m vs. controls	83.9%	75%	86.6%	P < 0.0001
> 12 m vs. controls	74.5%	63%	79.1%	P < 0.0001

*GR* generalization rate, *SS* sensitivity, *SC* specificity, *PT* permutation test, 0–6 m = post radiotherapy 0–6 months; 6–12 m = post radiotherapy 6–12 months; > 12 m = post radiotherapy > 12 months; vs versus

**Fig. 4:**
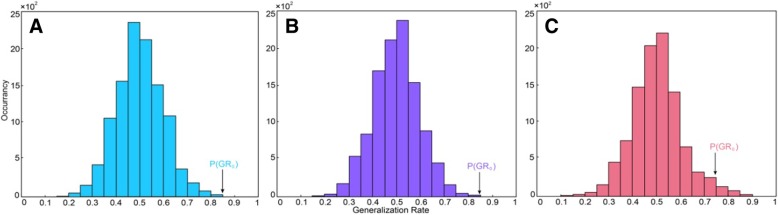
The permutation distribution of the estimate (repetition times: 10,000). **a** Post-RT 0–6 months versus the control classification. **b** Post-RT 6–12 months versus the control classification. **c** Post-RT > 12 months versus the control classification. X- and Y-labels represent the generalization rate and occurrence number, respectively. GR0 is the generation rate obtained by the classifier trained on the correct class labels

Whole brain WM connections. 0–6 months post-RT group and control group: the SVM classifier classification recognition rate reached 82.5% (SS = 83.3%, SC = 83.3%; *P* < 0.0001); 6–12 months post-RT group and control group: the SVM classifier classification recognition rate reached 78.4% (SS = 76.7%, SC = 76.7%; *P* < 0.0001); > 12 months post-RT group and control group: the SVM classifier classification recognition rate reached 76.3% (SS = 80%, SC = 80%; *P* < 0.0001) (Table 3). Compared with the control group, in each post-RT group, the brain WM connections (consistency) with discriminative ability were reduced (Figs. 6, 7 and 8).

**Table 3:** SVM classification results of white matter connections

	GR	SS	SC	Permutation test
0–6 m vs. controls	82.5%	83.3%	82.1%	*P* < 0.0001
6–12 m vs. controls	78.4%	76.7%	79.1%	*P* < 0.0001
> 12 m vs. controls	76.3%	80%	74.6%	*P* < 0.0001

*GR* generalization rate, *SS* sensitivity, *SC* specificity, *PT* permutation test; 0–6 m = 0–6 months post-RT; 6–12 m = 6–12 months post-RT; > 12 m = > 12 months post-RT; vs versus

**Fig. 5:**
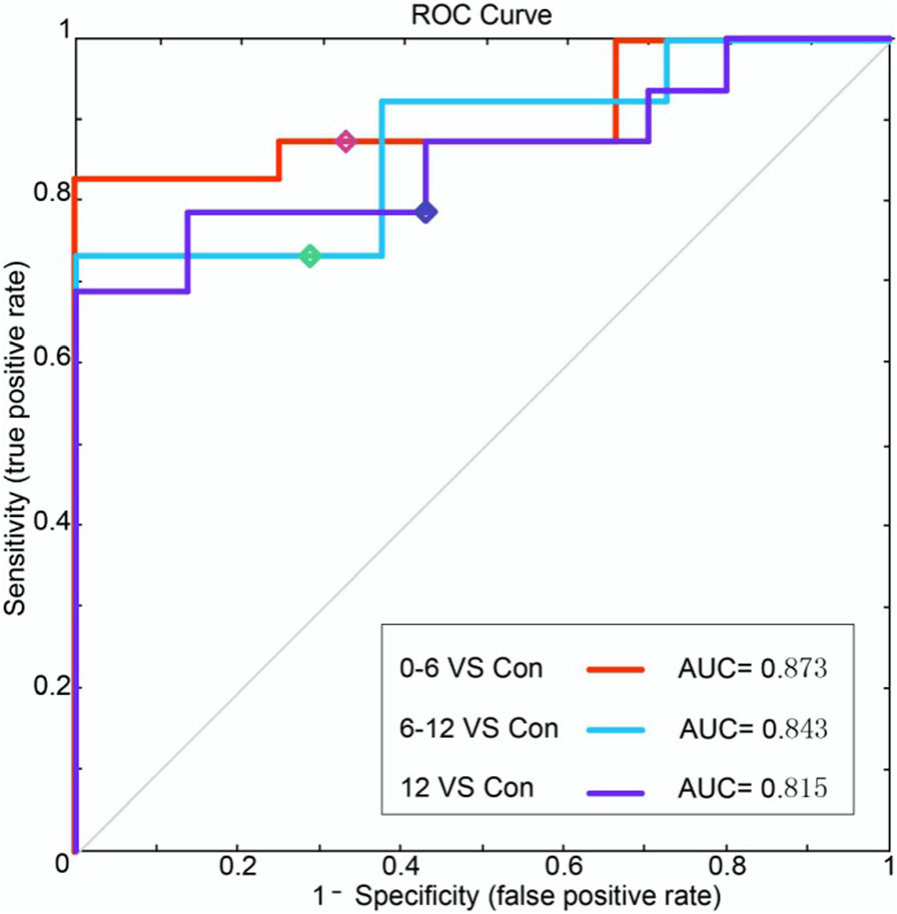
ROC curves of the SVM classifier. The orange broken line corresponds to the ROC curve of the SVM classifier for 0–6 months post-RT versus the control; the purple broken line, for 6–12 months post-RT versus the control; and the sky-blue real line, for > 12 months post-RT versus the control. A yellow, pea green or purple diamond on the curve corresponds to the classification rate, with zero as the classification threshold. AUC = area under the curve. This figure shows that our classifier had a better classification performance

**Fig. 6:**
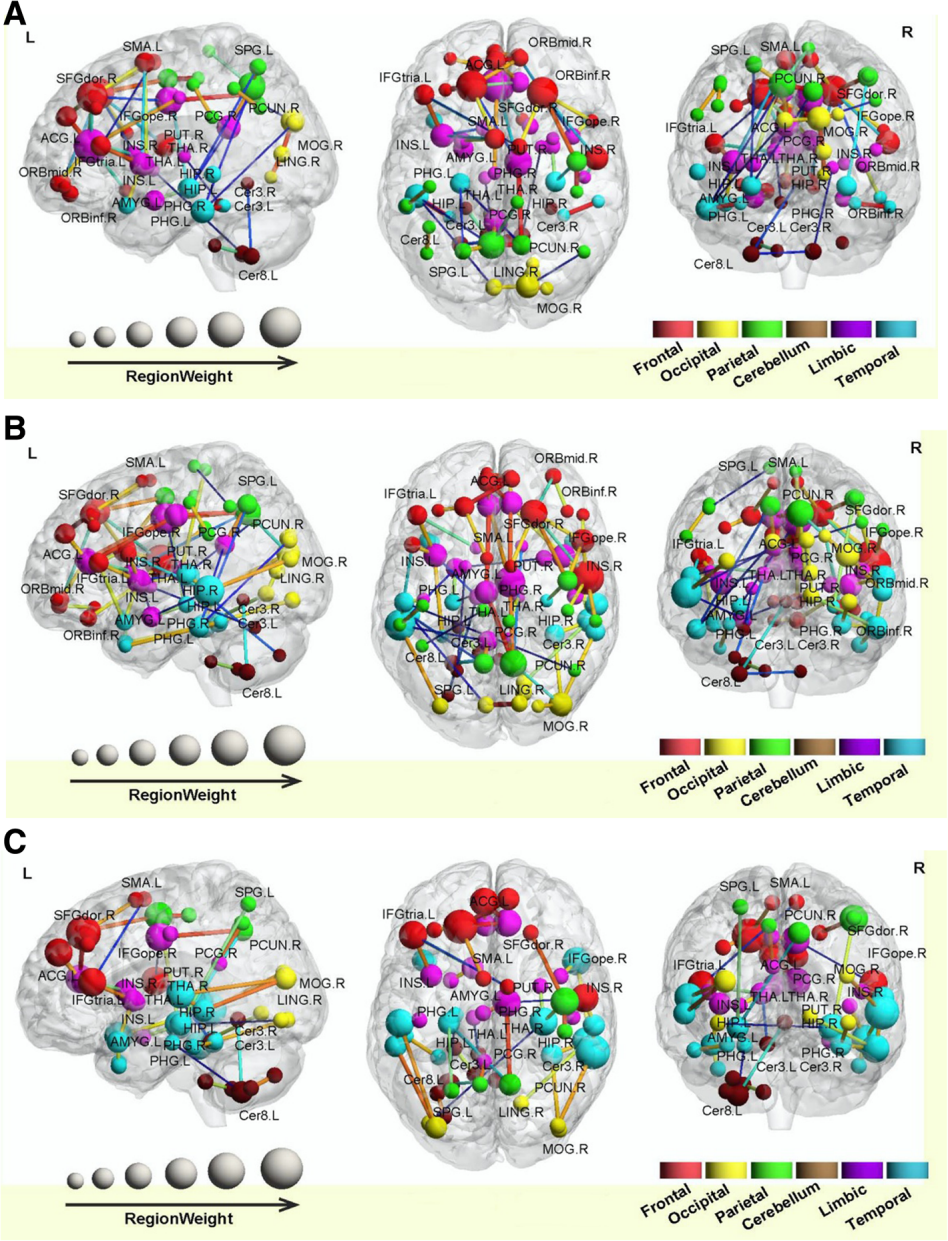
Region weights and distribution of the consensus WM connections. The classification for 0–6 (**a**), 6–12 (**b**) and > 12 months (**c**) post-RT versus the control is masked out. The thickness of the connections adjusts according to their connectivity strength. The connections are all decreased. The diameter of the sphere represents the corresponding weight of the region of interest (ROI). The ROIs are colour-coded according to brain areas (Red = Frontal cortex; Yellow = Occipital cortex; Green = Parietal cortex; Brown = Cerebellum; Purple = Limbic cortex; Sky blue = Temporal cortex). R = Right hemisphere, L = Left hemisphere. Med = Medial; Mid = Middle; Ope = Opercular; Tria = Triangular; Sup = Superior; SFG = Superior frontal; MFG = Middle frontal; IFG = Inferior frontal; ORB = Orbital frontal; TPO = Temporal pole; SMA = Supplementary motor area; SPG = Superior parietal; PoCG = Postcentral; PreCG = Precentral; PCUN = Precuneus; DCG = Middle cingulate; INS = Insula; ACG = Anterior cingulum; PCG = Post cingulum; CAU = Caudate; PUT = Putamen; STG = Superior temporal; MTG = Middle temporal; LING = Lingual; HIP = Hippocampus; ROL = Rolandic; VMS = Vermis; FFG = Fusiform; Cer = Cerebellum; PHG = Parahippocampal; PAL = Pallidum

**Fig. 7:**
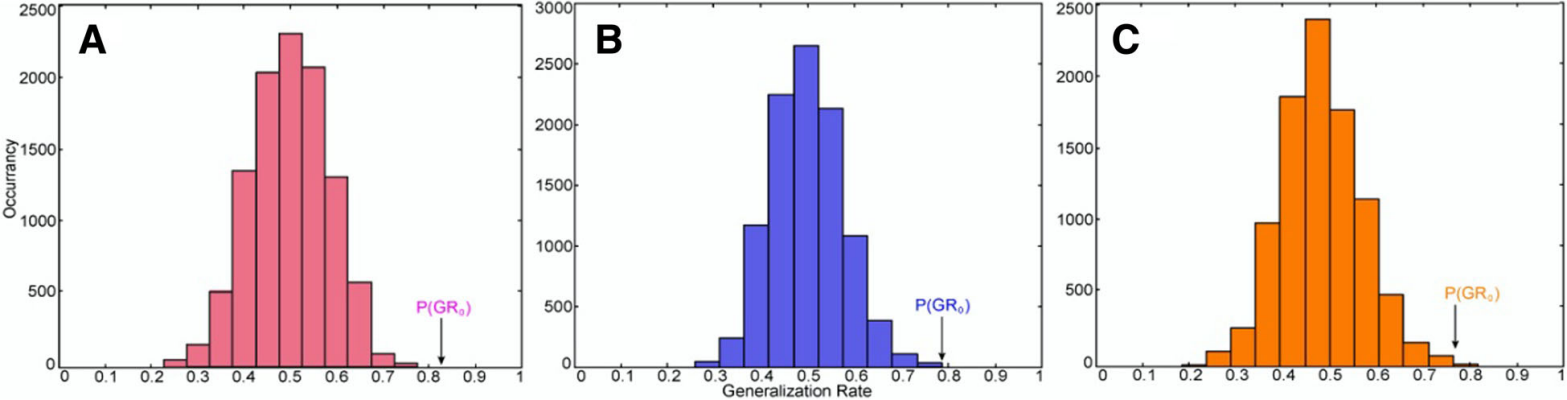
The permutation distribution of the estimate using an SVM with a Gaussian radial basis kernel function (repetition times: 10,000). **a** Classification of 0–6 months post-RT versus controls. **b** Classification of 6–12 months post-RT versus controls. **c** Classification of > 12 months post-RT versus controls. X- and Y-labels represent the generalization rate and occurrence number, respectively. GR0 is the generation rate obtained by the classifier trained on the correct class labels. With the generalization rate as the statistic, this figure reveals that the classifier learned the relationship between the data and the labels with a probability of being incorrect of 0.0001

**Fig. 8:**
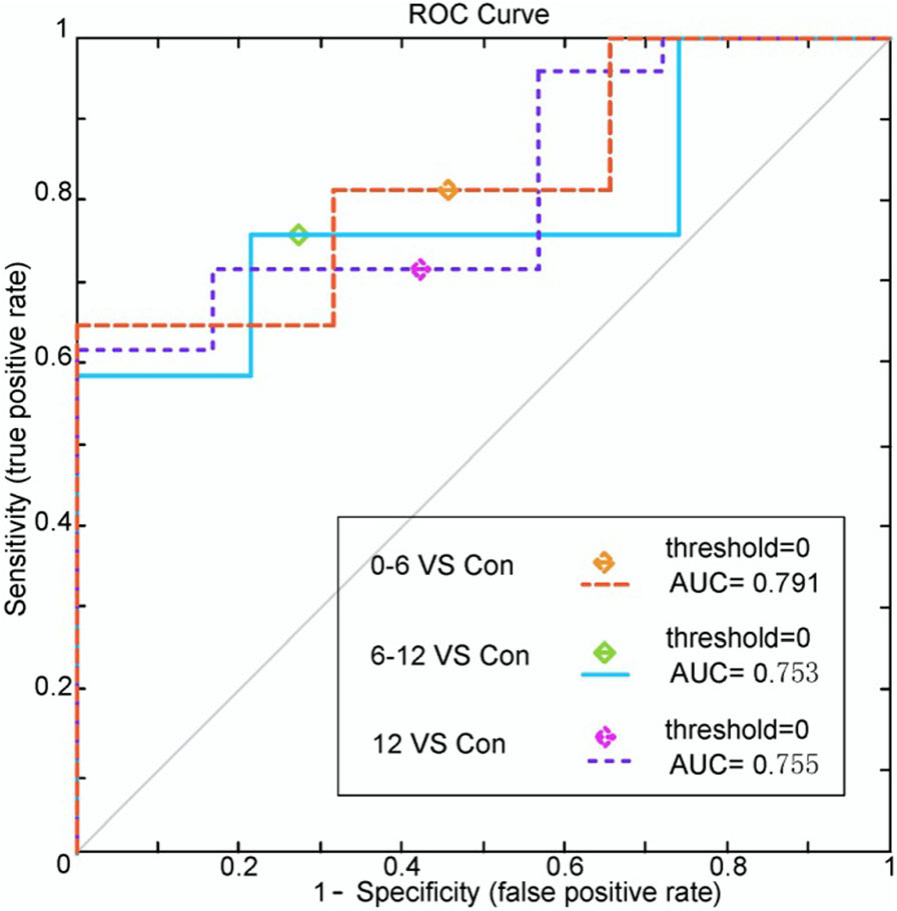
ROC curves of the SVM classifier. The orange broken line corresponds to the ROC curve of the SVM classifier for 0–6 months post-RT versus the control; the purple broken line, for 6–12 months post-RT versus the control; and the sky-blue real line, for > 12 months post-RT versus the controls. A yellow, pea green or purple diamond on the curve corresponds to the classification rate, with zero as the classification threshold. AUC = area under the curve. This figure shows that our classifier had a better classification performance

## Discussion

The present study is the first to use DTI-TBSS along with a machine learning method to explore dynamic changes in the whole brain WM microstructure and WM network. The results showed that the WM microstructure damage produced in NPC involved bilateral temporal lobe and bilateral cerebellar multiple brain regions, which had the ability to identify disease, and each of the three groups could be distinguished from the control group, achieving a higher recognition rate.

The FA values of the most disease-discriminating brain region were reduced, indicating that the WM integrity had been damaged. Previous studies have demonstrated that FA values are reduced significantly after brain RT [8, 19–21], and DTI could serve as a potential biomarker for the assessment of radiation-induced long-term white matter injury [22]. Brain oedema, demyelination of nerve fibres and destruction of myelin lead to a decrease in FA [9]. These WM microstructural changes were complex and dynamic. In the present study, with prolonged time after RT, the damage was aggravated, and the number of brain areas that had the most discriminative ability increased gradually. The most discriminating WM regions which underwent microstructural injury were mainly located in the temporal lobe and bilateral cerebella, probably because the regions were near the radiation fields and vulnerable to radiation damage. These results were consistent with previous research [11, 12]. However, in contrast to those reports, our results showed that the overall trend of radiation damage increased with the duration of RT rather than showing gradual recovery. At the early stage following RT, the injuries occurred in the bilateral cerebellum, and there was no significant damage in the temporal WM. Most likely, the integrity of the temporal WM did not appear to cause significant damage which could distinguish diseases early, the integrity of the temporal WM did not appeared significant damage, so that it did not have the ability to distinguish diseases at individual level. And the surrounding normal WM may have undergone a compensatory phenomenon, which offset some of the temporal WM damage. With the accumulation of time, the WM damage in the temporal lobe and cerebella gradually became significant, the number of the most discriminating WM regions increased, and the damage was most likely difficult to reverse. This may explain why radiation-induced encephalopathy has a long incubation period of up to 5.4 years [23]. From our results, we learned that WM injury is a gradual and irreversible process: the longer the time, the heavier the damage.

To a certain extent, the brain tissue could not repair or compensate for these injuries, which would lead to the occurrence of RBI. Previous studies showed that, in addition to injuring the temporal lobe, radiation induced extensive damage to the brain, including the frontal and parietal lobes [24, 25]. In the present study, there was no observation of WM damage in the extensive brain regions, possibly because in the machine learning method, the WM destruction in other brain regions did not achieve a higher ability to differentiate and identify RBI at the individual level. This is the advantage of the machine learning method relative to traditional statistical methods at the group level. The method can eliminate the interference caused by the differences in the sample size and the statistical approaches and then extract the most discriminating WM regions (which may be used as biomarkers of the disease), thereby facilitating clinical diagnosis and treatment. This research was the first time to introduce machine learning method into RBI of NPC, which is the innovation of this research. Most of the brain WM regions that were feasible for disease differentiation and identification appeared on the left side, which may be related to the more frequent occurrence of NPC on the left side in the present study.

According to reports in the literature, microscopic damage in normal-appearing WM, as indexed by lower FA, was related to poor intellectual outcome [26] and cognitive disability [27]. The decline in visual spatial executive ability, memory, attention, cognitive function, social function and auditory function may be related to the decreased FA value in the temporal gyrus WM [5, 28]. The decline in the ability to regulate cognitive function and memory may be related to the decreased FA value in the cerebellar hemisphere WM, and emotional disorders may be related to the decreased FA value in the WM of the vermis of the cerebellum [29, 30] and may explain the decline in NPC patient memory, visual space execution, hearing, naming function and significant anxiety and depression after RT [4, 7, 31]. The damage to the WM in these brain regions may be the pathological basis of neural network abnormalities, cognitive decline, and affective disorders.

A previous study used resting-state functional magnetic resonance imaging (rs-fMRI) to perform a short-term follow-up of 39 patients with a new diagnosis of NPC, and found that the brain function network connection pattern can be used as a biological marker to monitor RBI [7]. According to the idea of structure determining function, the most reliable way to assess brain function is to understand the anatomical structure of the brain and its potential brain circuits. Therefore, studying the anatomical network between regions of WM can supplement the understanding of pathophysiological mechanism of RBI, and it is of great significance to explore the neurological basis of cognitive dysfunction after RT. A study has also shown that the GM volume of NPC is significantly impaired after RT [25], and we speculated that WM connections between GM are significantly damaged. In this study, we used a machine learning method and DTI to explore the changes in the WM anatomic network in NPC patients after RT. The first step was using a deterministic fibre tracing method to reconstruct the fibre bundle and extracting the whole brain WM network. In the second step, we used the machine learning method to extract the most discriminating WM connections and explored the dynamic changes of the WM network in different periods following RT. The present study was the first to regard the brain WM connection pattern and its connection strength as a feature to classify NPC patients after RT, which is the innovation of this investigation.

Our study found that the WM network of NPC patients in different periods after RT had changed dynamically. Patients could be distinguished from normal controls based on the whole brain WM connection and achieved certain recognition rates, namely, 82.5, 78.4 and 76.3%, respectively. The strength of the most discriminating connection was reduced. Our results suggested that RBI can be regarded as an abnormal disease of the WM structural network. Perhaps the destruction of these structural networks and the reduction in their connections can explain some of the patients’ clinical manifestations and cognitive impairment.

These results, to a certain extent, further demonstrated that RBI is a disease related to the abnormal WM network of the whole brain. The high classification rate showed that a stable group difference between NPC patients after RT and the control group could be detected. The abnormal WM connection in these networks may be the cause of neuropsychological cognitive impairment in NPC patients. Within one year after RT, the number of WM connections with a significant reduction increased with time, and after a year, the number was reduced. This finding indicated that after a period of progressive aggravation, the destruction of the WM connection in the whole brain can be gradually restored, which may be related to the compensation and self-repair of the whole brain network. Perhaps, new brain WM connections are established. Brainstem dose is almost always a concern in radiation planning, in our analysis, the brainstem was included but changes in FA values were not significant. Previous research showed brainstem toxicity was reduced in patients treated with static Intensity-modulated radiotherapy (IMRT) (0.07%) and dynamic IMRT (0.08%) [32]. We speculated that due to the application of IMRT, the brain stem has been rarely damaged by radiation, which is consistent with previous research. These findings might provide therapeutic guidance for clinicians in radiation planning.

There are some limitations to this study. First, all of our NPC patients were treated with concurrent chemotherapy, and there was a certain difference in the dosage of the drug. How to exclude or quantify the effect of chemotherapeutic drugs on brain WM needs further study. Second, this study was a prospective cross-sectional study, and the next step requires a larger cohort study to explore changes in the dynamic effects of RT. Third, the present research lacked behavioural data. Next, we need to improve the mental and cognitive scale of subjects.

## Conclusion

In this study, the results revealed that groups in different periods post-RT and a control group can be distinguished from each other and can achieve a high recognition rate. The most serious and disease-discriminating brain regions were mainly located in the bilateral temporal and cerebellar WM, and the WM damage was gradually aggravated with time. At the same time, RBI was a disease exhibiting whole brain WM network anomalies. The strength of the consistent ability to discriminate disease (WM connections) was reduced and may cause cognitive dysfunction. Moreover, these brain discriminating WM regions and WM connection modes can supplement the understanding of RBI and can be used as biomarkers for the early clinical diagnosis of RBI.

## Abbreviations

DTI: Diffusion tensor imaging
FA: Fractional anisotropy
GM: Gray matter
GR: Generalization rate
NPC: Nasopharyngeal carcinoma
PT: Permutation test
RBI: Radiation brain injury
RT: Radiotherapy
SC: Specificity
SS: Sensitivity
SVM: Support vector machine
TBSS: Tract-based spatial statistic
WM: White matter

## References

[1] Jemal A, Bray F, Center MM, Ferlay J, Ward E, Forman D (2011). Global cancer statistics. CA Cancer J Clin.

[2] Leung SF, Kreel L, Tsao SY (1992). Asymptomatic temporal lobe injury after radiotherapy for nasopharyngeal carcinoma: incidence and determinants. Br J Radiol.

[3] Laack NN, Brown PD (2004). Cognitive sequelae of brain radiation in adults. Semin Oncol.

[4] Chen SC, Abe Y, Fang PT, Hsieh YJ, Yang YI, Lu TY (2017). Prognosis of hippocampal function after sub-lethal irradiation brain injury in patients with nasopharyngeal carcinoma. Sci Rep.

[5] Hsiao KY, Yeh SA, Chang CC, Tsai PC, Wu JM, Gau JS (2010). Cognitive function before and after intensity-modulated radiation therapy in patients with nasopharyngeal carcinoma: a prospective study. Int J Radiat Oncol Biol Phys.

[6] Greene-Schloesser D, Robbins ME (2012). Radiation-induced cognitive impairment--from bench to bedside. Neuro-Oncology.

[7] Qiu Y, Guo Z, Han L, Yang Y, Li J, Liu S (2018). Network-level dysconnectivity in patients with nasopharyngeal carcinoma (NPC) early post-radiotherapy: longitudinal resting state fMRI study. Brain Imaging Behav.

[8] King TZ, Wang L, Mao H (2015). Disruption of White matter integrity in adult survivors of childhood brain tumors: correlates with long-term intellectual outcomes. PLoS One.

[9] Kitahara S, Nakasu S, Murata K, Sho K, Ito R (2005). Evaluation of treatment-induced cerebral white matter injury by using diffusion-tensor MR imaging: initial experience. AJNR Am J Neuroradiol.

[10] Xie Y, Huang H, Guo J, Zhou D (2018). Relative cerebral blood volume is a potential biomarker in late delayed radiation-induced brain injury. J Magn Reson Imaging.

[11] Wang HZ, Qiu SJ, Lv XF, Wang YY, Liang Y, Xiong WF (2012). Diffusion tensor imaging and 1H-MRS study on radiation-induced brain injury after nasopharyngeal carcinoma radiotherapy. Clin Radiol.

[12] Xiong WF, Qiu SJ, Wang HZ, Lv XF (2013). 1H-MR spectroscopy and diffusion tensor imaging of normal-appearing temporal white matter in patients with nasopharyngeal carcinoma after irradiation: initial experience. J Magn Reson Imaging.

[13] Fang P, Zeng LL, Shen H, Wang L, Li B, Liu L (2012). Increased cortical-limbic anatomical network connectivity in major depression revealed by diffusion tensor imaging. PLoS One.

[14] An J, Fang P, Wang W, Liu Z, Hu D, Qiu S (2014). Decreased white matter integrity in mesial temporal lobe epilepsy: a machine learning approach. Neuroreport..

[15] Wang L, Shen H, Tang F, Zang Y, Hu D (2012). Combined structural and resting-state functional MRI analysis of sexual dimorphism in the young adult human brain: an MVPA approach. Neuroimage..

[16] Soussain C, Ricard D, Fike JR, Mazeron JJ, Psimaras D, Delattre JY (2009). CNS complications of radiotherapy and chemotherapy. Lancet..

[17] Roweis ST, Saul LK (2000). Nonlinear dimensionality reduction by locally linear embedding. Science..

[18] Dosenbach NU, Nardos B, Cohen AL, Fair DA, Power JD, Church JA (2010). Prediction of individual brain maturity using fMRI. Science..

[19] Nagesh V, Tsien CI, Chenevert TL, Ross BD, Lawrence TS, Junick L (2008). Radiation-induced changes in normal-appearing white matter in patients with cerebral tumors: a diffusion tensor imaging study. Int J Radiat Oncol Biol Phys.

[20] Prust MJ, Jafari-Khouzani K, Kalpathy-Cramer J, Polaskova P, Batchelor TT, Gerstner ER (2015). Standard chemoradiation for glioblastoma results in progressive brain volume loss. Neurology..

[21] Connor M, Karunamuni R, McDonald C, White N, Pettersson N (2016). Moiseenko, et al. dose-dependent white matter damage after brain radiotherapy. Radiother Oncol.

[22] Ravn S, Holmberg M, Sorensen P, Frokjær JB, Carl J (2013). Differences in supratentorial white matter diffusion after radiotherapy--new biomarker of normal brain tissue damage?. Acta Oncol.

[23] Chong VF, Fan YF, Mukherji SK (2000). Radiation-induced temporal lobe changes: CT and MR imaging characteristics. AJR Am J Roentgenol.

[24] Duan F, Cheng J, Jiang J, Chang J, Zhang Y, Qiu S (2016). Whole-brain changes in white matter microstructure after radiotherapy for nasopharyngeal carcinoma: a diffusion tensor imaging study. Eur Arch Otorhinolaryngol.

[25] Leng X, Fang P, Lin H, An J, Tan X, Zhang C (2017). Structural MRI research in patients with nasopharyngeal carcinoma following radiotherapy: a DTI and VBM study. Oncol Lett.

[26] Mabbott DJ, Noseworthy MD, Bouffet E, Rockel C, Laughlin S (2006). Diffusion tensor imaging of white matter after cranial radiation in children for medulloblastoma: correlation with IQ. Neuro-Oncology.

[27] Makale MT, McDonald CR, Hattangadi-Gluth JA, Kesari S (2017). Mechanisms of radiotherapy-associated cognitive disability in patients with brain tumours. Nat Rev Neurol.

[28] Breckel TP, Giessing C, Gieseler A, Querbach S, Reuter M, Thiel CM (2015). Nicotinergic modulation of attention-related neural activity differentiates polymorphisms of DRD2 and CHRNA4 receptor genes. PLoS One.

[29] Mormina E, Petracca M, Bommarito G, Piaggio N, Cocozza S, Inglese M (2017). Cerebellum and neurodegenerative diseases: beyond conventional magnetic resonance imaging. World J Radiol.

[30] Koziol LF, Budding D, Andreasen N, D'Arrigo S, Bulgheroni S, Imamizu H (2014). Consensus paper: the cerebellum's role in movement and cognition. Cerebellum..

[31] Ma Q, Wu D, Zeng LL, Shen H, Hu D, Qiu S (2016). Radiation-induced functional connectivity alterations in nasopharyngeal carcinoma patients with radiotherapy. Medicine (Baltimore).

[32] Sun PY, Chen YH, Feng XB, Yang CX, Wu F, Wang RS (2018). High-dose static and dynamic intensity-modulated radiotherapy combined with chemotherapy for patients with locally advanced nasopharyngeal carcinoma improves survival and reduces brainstem toxicity. Med Sci Monit.

